# Identification of endoplasmic reticulum stress-related signature characterizes the tumor microenvironment and predicts prognosis in lung adenocarcinoma

**DOI:** 10.1038/s41598-023-45690-3

**Published:** 2023-11-09

**Authors:** Li Wan, Zhike Chen, Jian Yang, Gaotian Wu, Yao Xu, Jian Cui, Xueping Zhao

**Affiliations:** 1 Soochow University Laboratory of Cancer Molecular Genetics, Medical College of Soochow University, Suzhou, China; 2 Department of Thoracic Surgery, The First Affiliated Hospital of Soochow University, Suzhou, China; 3 Department of Neurosurgery, The First Affiliated Hospital of Soochow University, Suzhou, China; 4 Department of Thoracic Surgery, Wuzhong District People’s Hospital, Suzhou, China; 5 School of Nursing, Medical College of Soochow University, Suzhou, Jiangsu China

**Keywords:** Non-small-cell lung cancer, Cancer microenvironment

## Abstract

Lung adenocarcinoma (LUAD) remains one of the most lethal malignancies worldwide, with a high mortality rate and unfavorable prognosis. Endoplasmic reticulum (ER) stress is a key regulator of tumour growth, metastasis, and the response to chemotherapy, targeted therapies and immune response. It acts via responding to misfolded proteins and triggering abnormal activation of ER stress sensors and downstream signalling pathways. Notably, the expression patterns of ER-stress-related-genes (ERSRGs) are indicative of survival outcomes, especially in the context of immune infiltration. Through consensus clustering of prognosis-associated ERSRGs, we delineated two distinct LUAD subtypes: Cluster 1 and Cluster 2. Comprehensive analyses revealed significant disparities between these subtypes in terms of prognosis, immune cell infiltration, and tumor progression. Leveraging the robustness of LASSO regression and Multivariate stepwise regression, we constructed and validated an ER Stress-associated risk signature for LUAD. This signature underwent assessments for its prognostic value, correlation with clinical attributes, and interaction within the tumour immune microenvironment. By integrating this signature with multivariate cox analysis of distinct pathological stages, we devised an enhanced nomogram, validated through various statistical metrics, with an area under the curve for overall survival at 1, 3, and 5 years post-diagnosis being 0.79, 0.80, and 0.81, respectively. In conclusion, our findings introduce a composite signature of 11 pivotal ERSRGs, holding promise as a potent prognostic tool for LUAD, and offering insights for immunotherapeutic and targeted intervention strategies.

## Introduction

Lung cancer persistently ranks among the top malignancies in terms of prevalence and fatality, representing a global health challenge. In 2022, a staggering 236,000 new lung cancer cases were recorded globally, with a disheartening overall survival rate of just 22%^1,2^. Remarkably, this formidable disease reigns as the predominant cancer in 37 countries, with China grappling with its prevention and management due to prevalent risk factors such as high smoking rates and substantial passive smoke exposure^2,3^. Within this, Lung adenocarcinoma (LUAD) emerges as the primary subtype, constituting 38.5% of all lung cancer variants^4^. Despite concerted research efforts to identify therapeutic targets for LUAD, challenges like resistance to targeted therapies and insidious metastases compromise treatment outcomes. This underscores the pressing need to unearth novel therapeutic targets and prognostic markers that can refine LUAD management and prognostication.

The endoplasmic reticulum (ER), an intricate network of branching tubules and flattened sacs pervading the cytosol, plays a pivotal role in eukaryotic cell function. It orchestrates a gamut of cellular processes, from protein synthesis and lipid production to calcium ion sequestration^5,6^. However, the ER’s functionality is contingent on its stringent quality control mechanisms. Disruptions, such as protein misfolding, slow folding kinetics, accumulation of non-native proteins, or calcium ion dysregulation due to membrane breaches, activate the ER’s three-pronged unfolded protein response (UPR) system^6^. This system, comprising the protein kinase R (PKR)-like endoplasmic reticulum kinase (PERK), inositol-requiring enzyme 1 (IRE1), and activating transcription factor 6 (ATF6) pathways, institutes measures to degrade ER-associated aberrant proteins, thereby reinstating ER homeostasis^7,8^. This intricate cellular countermeasure is termed ER stress. A successful, non-lethal ER stress response can rejuvenate ER stability, fortifying cellular resilience against stressors and bolstering survival. Conversely, unmitigated or excessive ER stress precipitates cell death^7^.

The nexus between ER stress and LUAD remains enigmatic, with studies presenting contrasting views. A majority postulate that ER stress curtails LUAD progression by instigating apoptosis^9–11^. In contrast, others contend that ER stress might inadvertently fuel processes like epithelial-mesenchymal transition (EMT) and bolster LUAD cell migration^12^. Collectively, these findings underscore the multifaceted role of ER stress in LUAD, accentuating its potential as a therapeutic fulcrum. Thus, a holistic, systematic exploration of the ER stress-LUAD relationship is imperative.

## Results

### Identification of two distinct molecular isoforms based on ER stress-related genes in patients of LUAD

**Figure 1:**
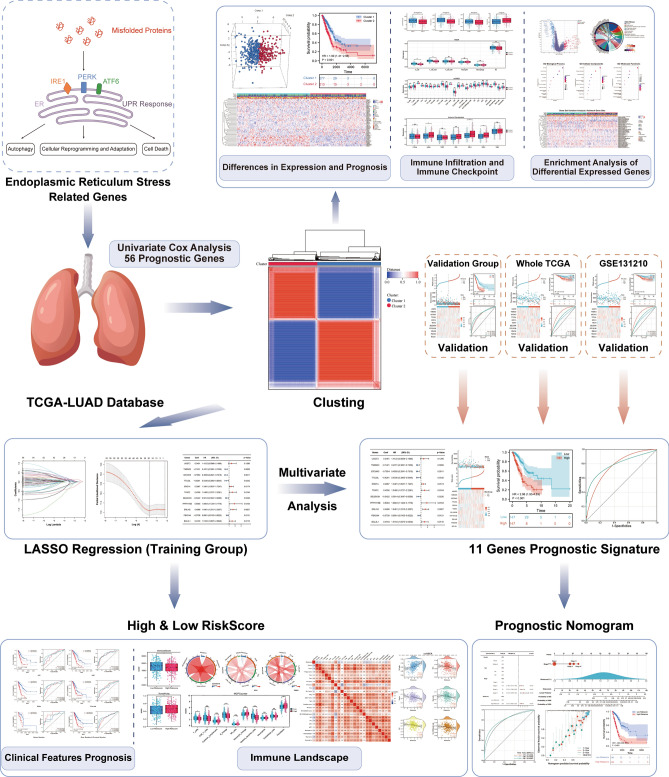
Flowchart of the prognostic model development and subsequent analyses in LUAD. The process initiates with the identification of prognostically significant genes from the pool of ER-stress-related-genes (ERSRGs) using COX regression. This foundational step facilitates the creation of a robust prognostic model via consensus clustering, segregating into two distinct molecular subgroups. With the model established, a series of in-depth analyses ensue, including differential gene expression, immune infiltration, functional enrichment, and clinical correlations, culminating in the formulation of an optimized nomogram for LUAD prognosis.

**Figure 2:**
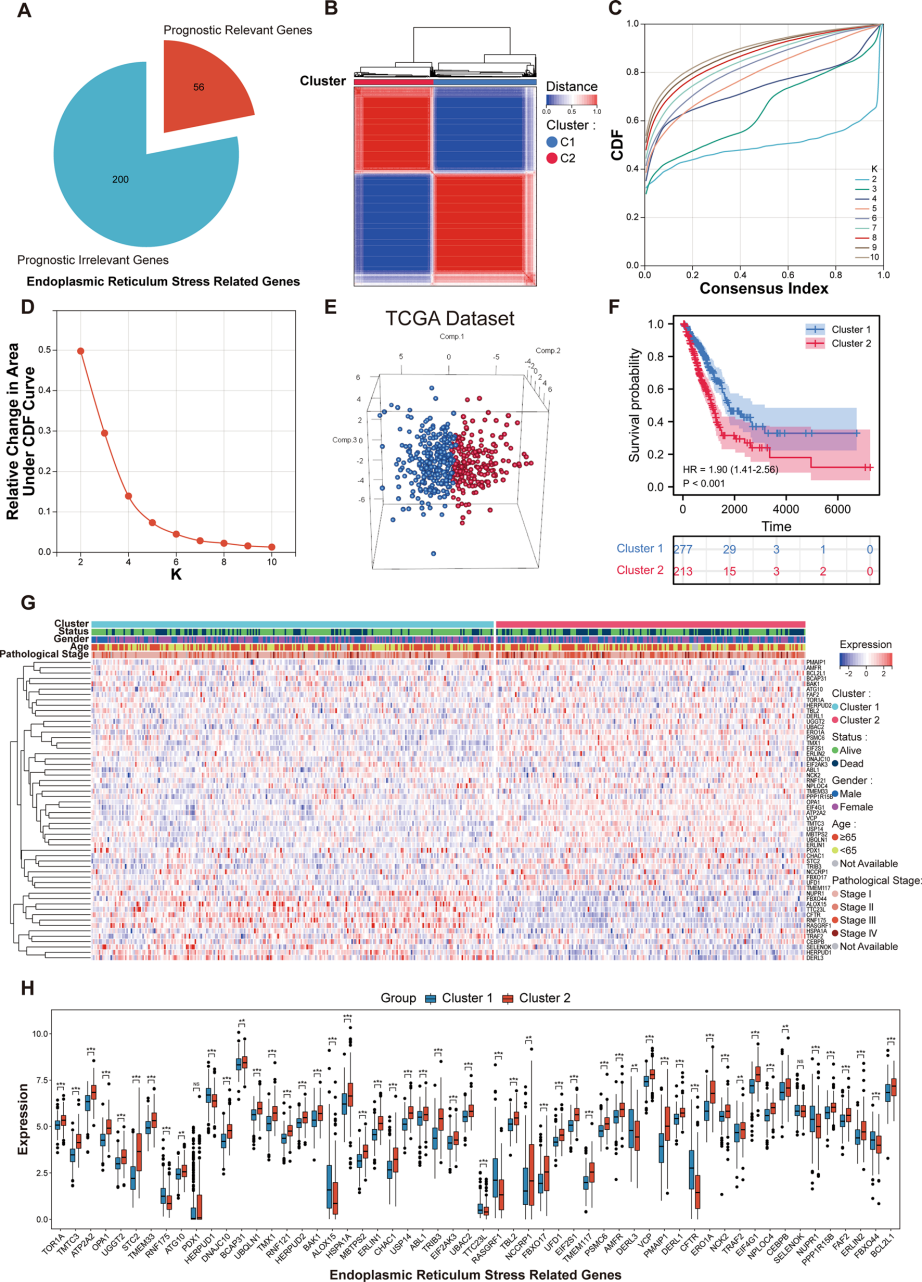
Unveiling endoplasmic reticulum stress-associated subtypes through consensus clustering and subsequent analyses. (**A**) Pie chart visualization of the results from a one-way cox analysis conducted on 256 ERSRGs, with a distinct subset of 56 genes emerging as significantly associated with patient survival. (**B**–**D**) In-depth consensus cluster analysis, applied to these 56 pivotal genes derived from a pool of 490 LUAD samples, with an optimal cluster number at k = 2, highlighting the formation of two distinct molecular subtypes. (**E**) Three-dimensional Principal Component Analysis (3D PCA) vividly delineating the segregation between Cluster 1 and Cluster 2, providing a spatial visualization of the distinct molecular landscapes. (**F**) Kaplan–Meier survival analysis curves showcasing the distinct overall survival (OS) trajectories for patients in both identified subgroups. (**G**) Heatmap showcasing the expression levels of the 56 ERSRGs across the two subtypes, emphasizing the molecular heterogeneity between the clusters. It was generated using the 'gplots' package (version 3.1.3) in R (version 4.2.1, [https://www.r-project.org/]). (**H**) Histogram representing the contrasting expression patterns of the 56 ERSRGs between the two subtypes. Created using the 'ggpubr' package (version 0.6.0) and 'ggplot2' package (version 3.4.2) in R.

Figure 1 provides an overarching blueprint of our methodological approach in this study, encapsulating the journey from the initial identification of ER-stress-related genes to the eventual construction of an ER Stress-related prognostic model for LUAD and its subsequent analyses. Diving deeper into our analytical journey as showcased in Fig. 2, we meticulously explored the dataset. Starting with a comprehensive pool, 256 genes were sourced from the GOBP_RESPONSE_TO_ENDOPLASMIC_RETICULUM_STRESS gene set, marking them as ER-stress-related-genes (ERSRGs). Through the precise lens of univariate cox regression analysis, this list was refined to 56 genes with pronounced prognostic implications, as depicted in Fig. 2A. The expression profiles of these genes, when applied to the LUAD patient samples from the TCGA cohort, directed us towards consensus clustering. This bifurcated our dataset into two distinct molecular subtypes, optimally represented at cluster number K = 2 (Fig. 2B–D). The robustness of this clustering was further echoed by a principal component analysis (PCA), vividly segmenting the samples into Cluster 1 and Cluster 2, as depicted in Fig. 2E. Upon further analysis, we identified pronounced differences in prognosis between the two clusters. Notably, patients within Cluster 2 demonstrated a significantly reduced survival rate, as evident in Fig. 2F. This poor prognosis in Cluster 2 was further underscored by its association with more advanced pathological stages. Specifically, Cluster 2 had a notably higher representation of patients in Pathological Stages III and IV. Furthermore, this cluster was characterized by elevated counts of Tumour grades T3 and T4, indicating more aggressive tumour growth, and instances of Metastasis grade M1, suggesting potential spread to distant organs. The advanced nodal involvement was also evident with heightened counts of Node stages N1 and N2. These clinical attributes are comprehensively illustrated in Supplementary Fig. S1. In a further exploration of the molecular distinctions between the clusters, we analyzed the expression profiles of the 56 ERSRGs. Our analysis revealed that Cluster 2 predominantly exhibited heightened gene expressions, suggesting a more aggressive molecular phenotype. This differential gene expression between the clusters is clearly visualized in the heatmap and bar graph representations, as demonstrated in Fig. 2G,H.

### Different immune landscapes were exhibited by two subtypes of patients with LUAD

**Figure 3:**
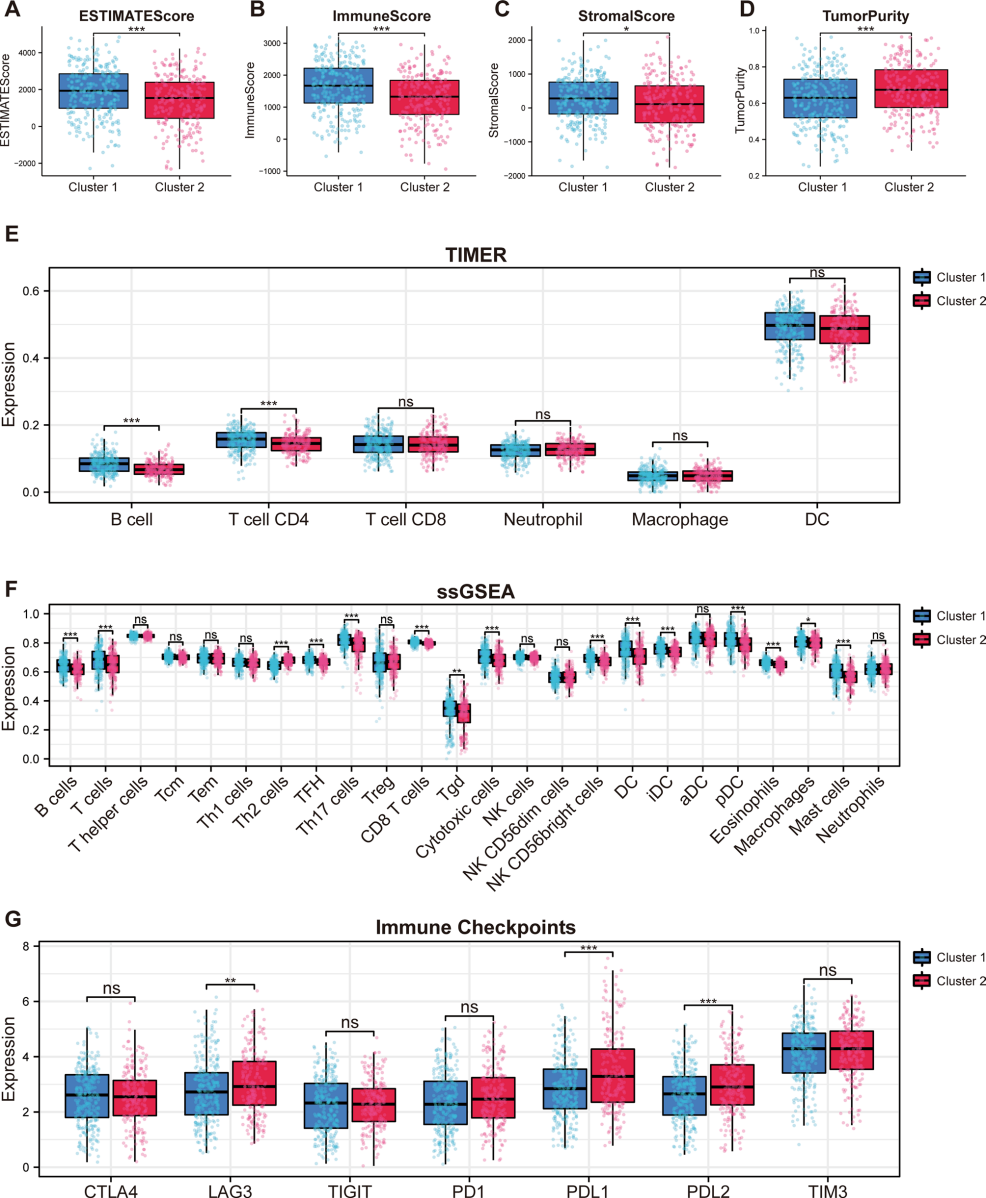
Comprehensive immune landscape and checkpoint expression analysis across the two LUAD subtypes. (**A**–**D**) Comparative depiction of the immune and stromal components in the tumor microenvironment, showcasing ESTIMATEScore, ImmuneScore, StromalScore, and TumorPurity, all derived using the ESTIMATE algorithm. The figures highlight the pronounced differences in the immune landscape between the two clusters. (**E**) Immune cell infiltration analysis for the two subtypes using the TIMER algorithm, delineating the distribution and abundance of six immune cell types across the clusters. (**F**) Comparative immune infiltration analysis between the two subgroups, utilizing the ssGSEA algorithm. The representation details the activity and presence of 24 distinct immune cell types, highlighting the differences in immune cell dynamics between Cluster 1 and Cluster 2. (**G**) Comparative analysis illustrating the differential expression patterns of key immune checkpoints between the two subtypes. The representation underscores the distinct molecular landscapes, emphasizing the variations in markers pivotal for modulating the immune response in Cluster 1 and Cluster 2.

To understand the immunological disparities between the two clusters, we deployed a series of analytical algorithms. Utilizing the ESTIMATE algorithm, it was evident that Cluster 2 presented a distinct immune profile characterized by diminished ESTIMATEScore, ImmuneScore, and StromalScore, coupled with an enhanced TumourPurity, as illustrated in Fig. 3A–D. Delving deeper with the TIMER algorithm, our data highlighted a reduced abundance of B Cells and CD4+ T Cells in Cluster 1, a trend presented in Fig. 3E. Broadening our scope with the ssGSEA algorithm to encapsulate the overall immune infiltration landscape, we discerned stark contrasts between the clusters. Notably, Cluster 2 showcased augmented immune activities in a plethora of cell types, including B Cells, T Cells, TFH, Th17 Cells, CD8 T Cells, Tgd, Cytotoxic Cells, NK CD56 bright Cells, DCs, iDCs, pDCs, Eosinophils, Macrophages, and Mast Cells. In contrast, Cluster 1 dominated in the immune activity of TH2 Cells, as captured in Fig. 3F. Our investigation did not stop there. When examining the expression of crucial immune checkpoints, we discovered that markers pivotal for immune modulation, namely LAG3, PD-L1, and PD-L2, were significantly upregulated in Cluster 2, as seen in Fig. 3G. This comprehensive analysis suggests that the immune environment of Cluster 2 might be more skewed towards facilitating tumor immune evasion, underscoring the potential therapeutic implications.

### Assessment of differential expression and functional enrichment analysis of genes in two subtypes

**Figure 4:**
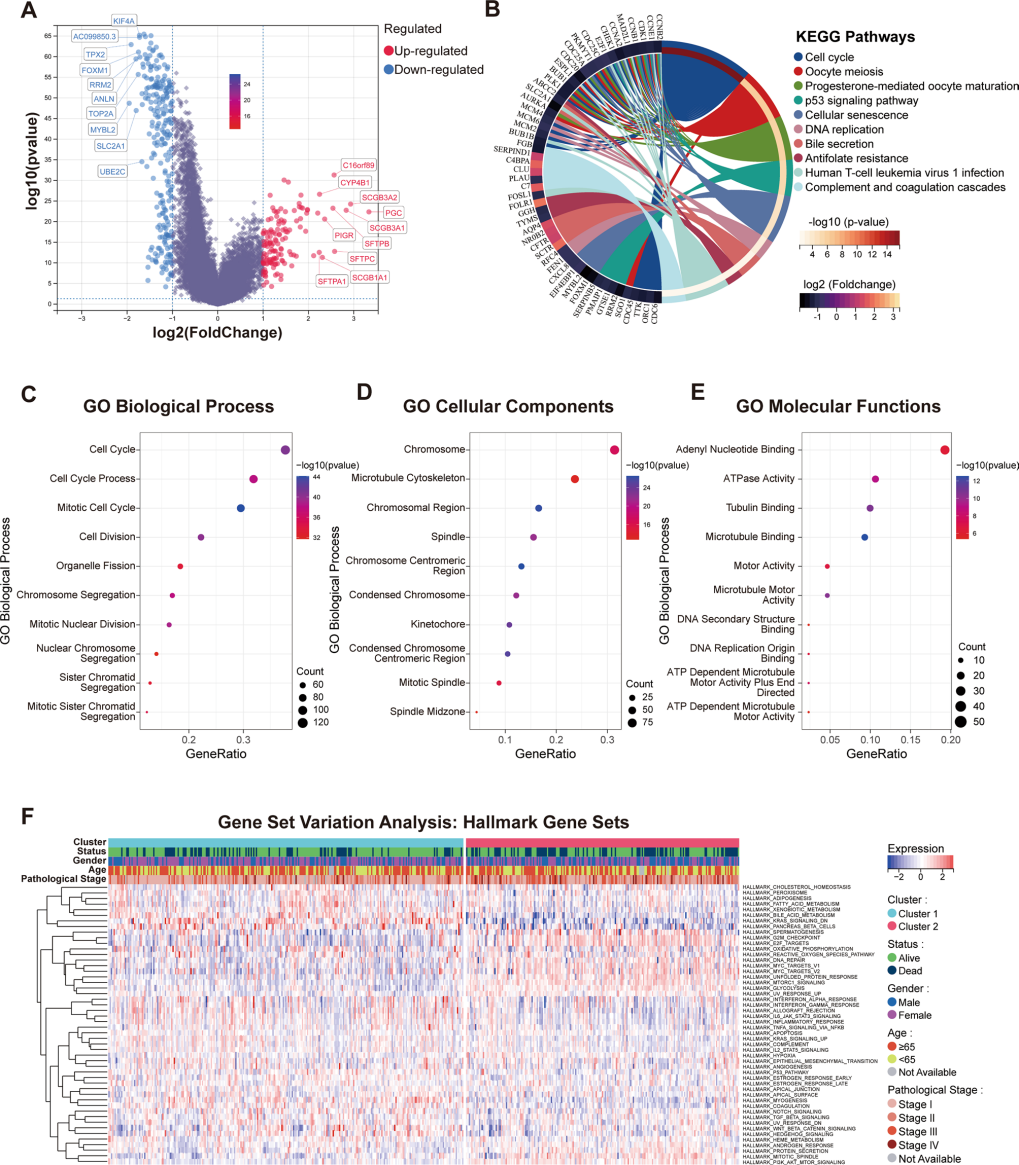
Comprehensive functional enrichment analysis highlighting distinct molecular landscapes of the two subtypes. (**A**) A detailed volcano plot visualizing the differential gene expression landscape between Cluster 1 and Cluster 2, sourced from the TCGA database, shedding light on the scale and significance of these variations. (**B**) KEGG pathway enrichment analysis of genes that displayed substantial differences in expression levels (criterion: |FoldChange|> 2 and *p* < 0.05), unveiling key biological pathways that differentiate the subtypes. (**C**–**E**) Gene Ontology (GO) analysis, dissecting the roles of the differentially expressed genes (|FoldChange|> 2 and *p* < 0.05) across three dimensions: Biological Process, Cellular Components, and Molecular Functions, providing insights into the underlying functional mechanisms. (**F**) Heatmap representation illustrating the GSVA scores for the two subtypes, capturing the breadth and depth of pathway activities and underlining the distinct enrichment landscape of Cluster 1 and Cluster 2. The heatmap was generated using the 'gplots' package (version 3.1.3) in R (version 4.2.1, [https://www.r-project.org/]).

Employing the R package ‘limma’ (version 3.40.6), we embarked on a rigorous differential analysis between Cluster 1 and Cluster 2. The resultant distinctions in gene expression, including both up- and down-regulated genes, were intuitively visualized using a volcano plot, adhering to a stringent fold-change criterion (|FoldChange| >2) as showcased in Fig. 4A. To decipher the underlying biological intricacies behind these disparities, a functional enrichment analysis was initiated. Our differential analysis yielded 379 significantly varied genes, which we then subjected to KEGG pathway enrichment. This analysis unmasked that the differentially expressed genes predominantly mapped to critical pathways associated with cell cycle dynamics, encompassing Oocyte meiosis, p53 signalling pathway, DNA replication, and Cellular senescence (Fig. 4B). Intriguingly, additional biological processes like Progesterone-mediated oocyte maturation and Bile secretion were also found to be distinctly regulated between the subtypes. Further enriching our understanding, we employed GO analysis on these differentially expressed genes, aiming to pinpoint their predominant involvements in Biological Process, Cellular Components, and Molecular Functions. As delineated in Fig. 4C–E, a recurring theme emerged, spotlighting an abundance of genes associated with cell-cycle-centric processes and components, notably, Cell Cycle Process and DNA Replication Origin Binding. To provide a more holistic view of the enrichment landscape, the GSVA algorithm was harnessed. This exploration identified 50 statistically significant biological pathways, which are vividly captured in a heatmap representation in Fig. 4F. Distinctly, Cluster 2 emerged as potently aligned with oncogenic processes, suggesting its potential role in driving malignant transformation and prognosis through modulations in cell cycle and proliferative dynamics.

### Construction of prognostic signature of ERSRGs using the LUAD database from TCGA

**Figure 5:**
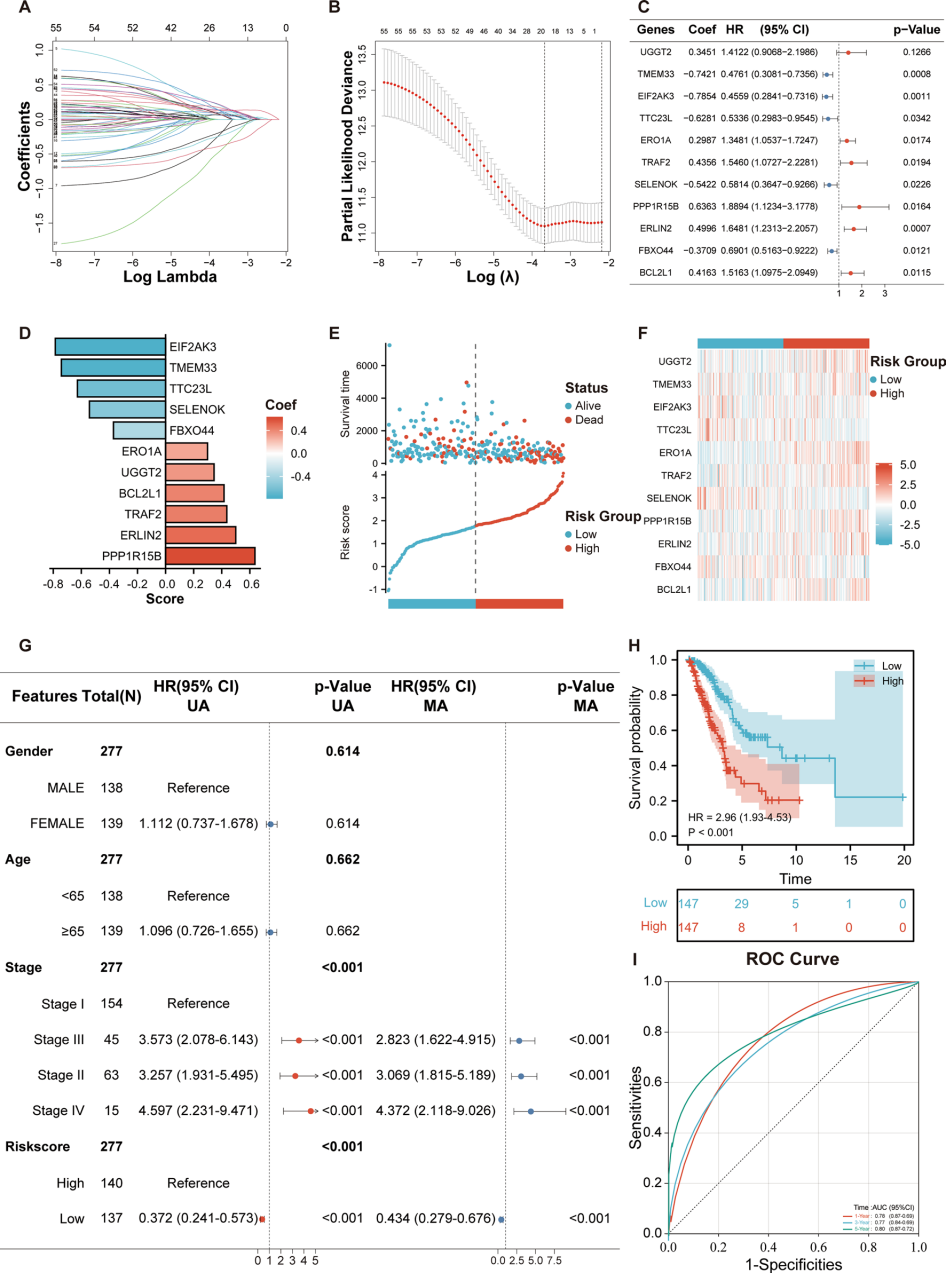
Construction of the ERSRGs Prognostic Signature in LUAD. (**A**, **B**) LASSO regression analysis visualized, pinpointing the most salient among the 56 ERSRG prognosis-associated genes, emphasizing the regularization and feature selection process. (**C**) A subsequent Multivariate Cox analysis on the 19 genes derived from LASSO, narrowing down to 11 genes with significant prognostic implications. (**D**) Bar chart representation of the 11 genes' Coef values, which formed the core of our prognostic signature. (**E**) Scatter plot elucidating the survival disparity between the high and low-risk groups within the training set, accentuating the risk-score's potency in stratifying patient outcomes. (**F**) Visual depiction of gene expression variations through a heat map, emphasizing the differential expression patterns of the 11 signature genes between the high and low-risk groups. This visualization was crafted with the assistance of the 'gplots' package (version 3.1.3) in R (version 4.2.1, [https://www.r-project.org/]). (**G**) Multivariate Cox analysis juxtaposing risk scores with clinical factors, corroborating the risk score's capacity as an independent prognostic determinant. (**H**) Kaplan–Meier overall survival curves, delineating the stark survival differential between LUAD patients stratified by their risk scores based on the prognostic signature. UA: Univariate Analysis, MA: Multivariate Analysis. (**I**) Time-dependent ROC curves, encapsulating the signature's predictive accuracy across 1-, 3-, and 5-year intervals within the TCGA cohort.

Recognizing the pivotal role of ERSRGs in LUAD progression, we endeavored to devise a prognostic risk model to discern its potential in forecasting patient outcomes. To bolster the model’s robustness, the cohort of 499 LUAD patients from TCGA was judiciously split into training and validation sets, ensuring a balanced representation. Initiating our model’s construction, we employed LASSO-Cox regression on the 56 prognosis-associated genes within the training cohort. This rigorous analytical approach, designed to mitigate overfitting in intricate datasets, culminated in the selection of 19 pivotal genes, as visualized in Fig. 5A,B. Delving deeper, a subsequent multivariate stepwise regression refined our gene list further, culminating in 11 genes that stood out as potential prognostic markers. Their significance and impact are delineated in a forest plot (Fig. 5C) with corresponding Coef values vividly portrayed in a bar chart (Fig. 5D). Upon obtaining the Coef values, we calculated the Risk-Score for each sample by multiplying each gene’s expression with its respective Coef. Based on these scores, we stratified the cohort into high-risk and low-risk factions. A striking divergence in outcomes became evident: the high-risk faction grappled with increased mortality, while the low-risk group showcased enhanced survival prospects, as illustrated in Fig. 5E. A heatmap further emphasized the differential expression of these 11 genes across the risk categories, underscoring their prognostic relevance (Fig. 5F). Our analytical rigor continued with a multivariate Cox regression, which, post the incorporation of significant clinical variables (*p* < 0.1) from the training cohort, affirmed the risk score’s stature as a standalone prognostic determinant (Fig. 5G). Reinforcing this notion, Kaplan-Meier survival curves, stratified by the risk score, spotlighted the compromised survival of the high-risk group (Fig. 5H). Finally, the model’s predictive prowess was validated via a time-dependent ROC curve for the training set, achieving impressive survival rate accuracies of 0.70, 0.77, and 0.75 for 1-, 3-, and 5-year intervals respectively (Fig. 5I).

### Validation of the prognostic signature

**Figure 6:**
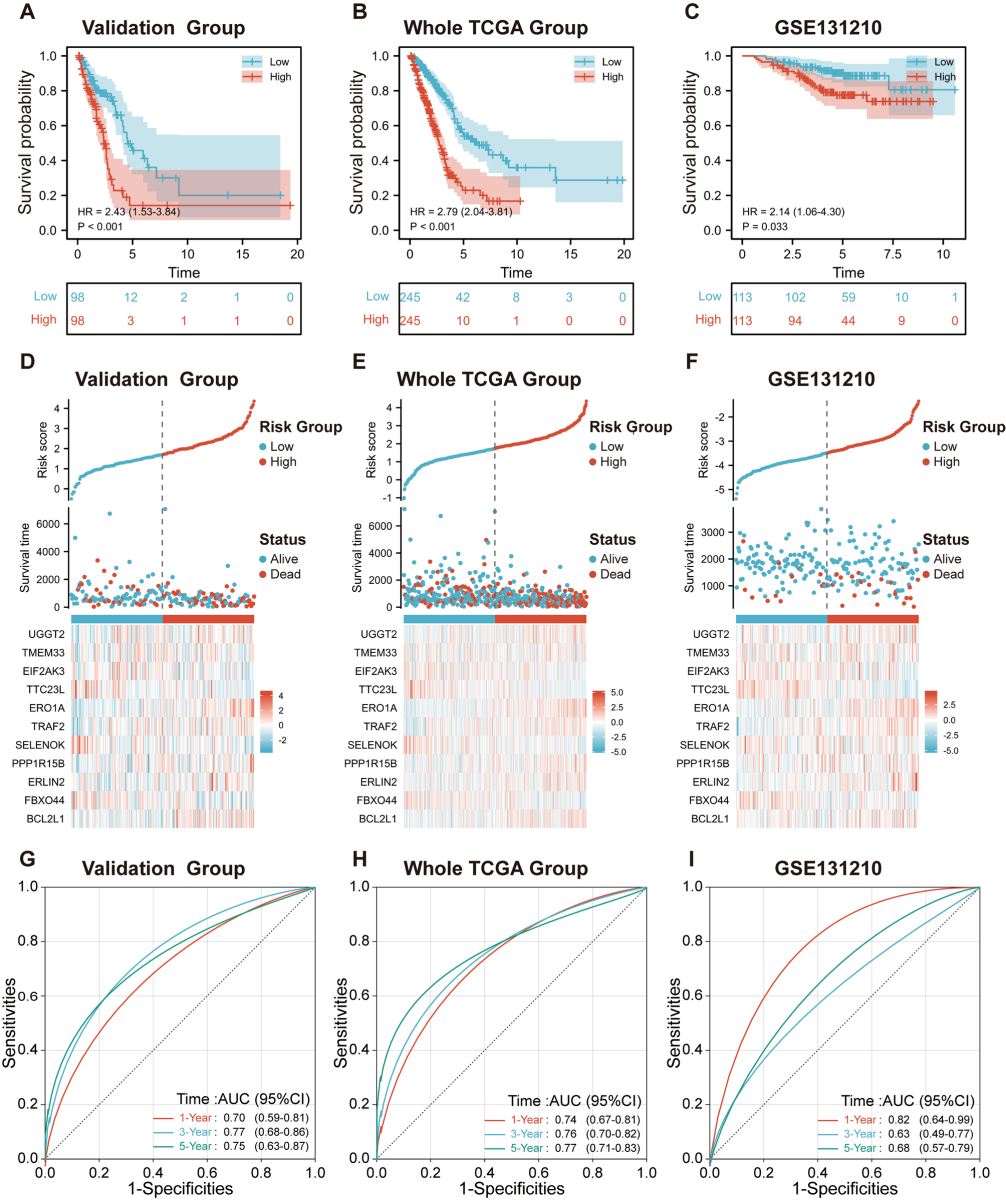
Comprehensive validation of the prognostic signature across diverse datasets. (**A**–**C**) Kaplan–Meier survival curves delineating the overall survival distinction between high and low Risk-Score groups within the validation set, the entire TCGA cohort, and the GSE131210 dataset. (**D**–**F**) Plots delineating the patients' outcome trajectories according to Risk-Score within the validation cohort. Alongside, heat maps showcase the transcriptional nuances of the signature genes across high and low-risk categories. The risk distribution was visualized using the 'ggplot2' package (version 3.4.2, [https://ggplot2.tidyverse.org/]), while the heatmap was generated with the 'gplots' package (version 3.1.3) in R (version 4.2.1, [https://www.r-project.org/]). (**G**–**I**) Time-dependent ROC curves elucidating the predictive efficacy of the Risk-Score for 1-, 3-, and 5-year overall survival durations across the validation cohort, with specific AUC values underscoring the signature's robustness in prognosis prediction.

Upon meticulously crafting a survival model anchored on 11 pivotal genes, it was imperative to rigorously validate its prognostic prowess across diverse datasets to cement its reliability and applicability. The validation phase was orchestrated in three distinct cohorts: the validation set, the holistic TCGA database, and the external GSE131210 dataset. Leveraging the Coef values derived from the training cohort, we calculated the risk scores for patients in the aforementioned datasets. Employing the median risk score as a threshold, we stratified the LUAD patients into high-risk and low-risk factions. A conspicuous trend emerged across all cohorts: the high-risk group invariably grappled with compromised survival outcomes. Kaplan–Meier survival curves for each cohort distinctly illustrated this survival disparity, with p-values underscoring the statistical significance (Validation Group: *p* < 0.001, Whole TCGA Group: *p* < 0.001, GSE131210: *p* = 0.033; Fig. 6A–F). To further underscore the signature’s prognostic veracity, we conducted time-dependent ROC analysis. The ensuing curves, encapsulated in Fig. 6G–I, exhibited commendable predictive prowess across distinct time intervals. Specifically, for the validation group, the 1-year, 3-year, and 5-year ROC values were 0.7, 0.77, and 0.75, respectively. Within the entire TCGA cohort, the corresponding values stood at 0.74, 0.76, and 0.77. The external GSE131210 dataset showcased a slightly varied pattern with values of 0.82 for the 1-year, 0.63 for the 3-year, and 0.68 for the 5-year. These metrics further reinforced the signature’s prognostic acumen, underscoring its consistency and reliability across different datasets and timeframes. In summation, our assiduously crafted prognostic model, embodying 11 ERSRGs, emerged as a highly precise and informative tool for discerning the risk and prognosis trajectory of LUAD patients. The robust validation across disparate datasets underpins its potential as a reliable prognostic asset in the clinical setting.

### Correlation of prognostic features of patients with LUAD with clinical characteristics

**Figure 7:**
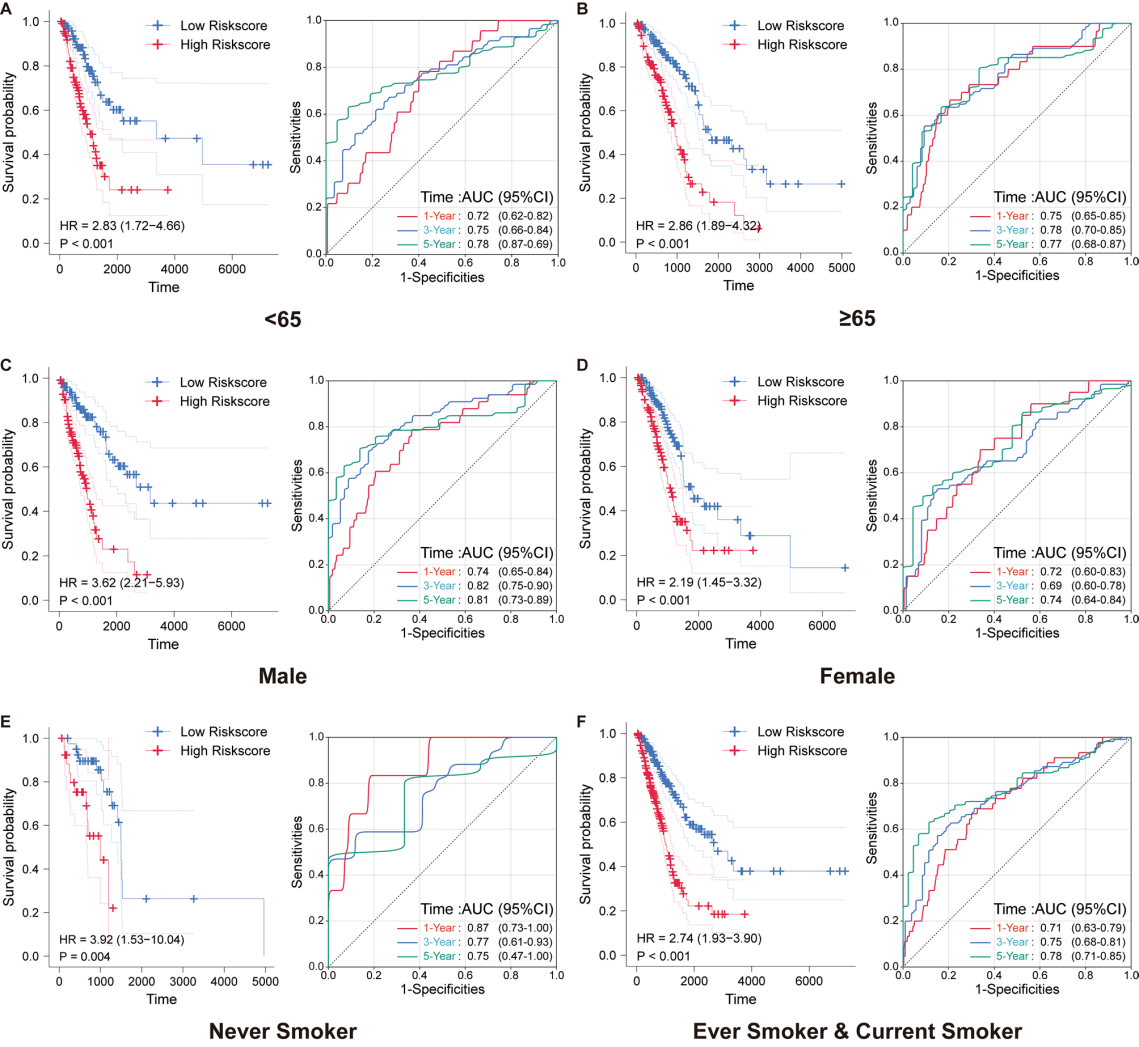
Prognostic validation across diverse demographic and clinical spectrums. (**A**, **B**) Illustrate the ROC curves at 1-, 3-, and 5-year intervals for patients below and above 65 years of age, with AUC values revealing a consistent prognostic performance. (**C**, **D**) Depict the gender-based analysis, where male and female patients exhibit distinctive ROC curves, underlining the model's predictive acumen across genders. (**E**, **F**) Emphasize the model's prognostic validity across smoking statuses, highlighting the AUC values for 'Never Smoker' and 'Ever Smoker & Current Smoker' categories, affirming the model's robust predictive capability across a pivotal lifestyle determinant. Each subfigure underscores the prognostic model's robustness, offering nuanced insights into its predictive validity across varied demographic and clinical scenarios.

To elucidate the clinical implications of our constructed prognostic model, we scrutinized its performance against diverse clinical characteristics derived from the TCGA database. A consistent pattern emerged: LUAD patients categorized under the high-risk group consistently displayed more adverse clinical attributes compared to their counterparts in the low-risk group, irrespective of the clinical parameters considered. This trend is graphically captured in Supplementary Fig. S2. Delving deeper into the time-dependent ROC analyses, a detailed examination of the ROC curves across varying patient demographics and clinical parameters illuminated the prognostic model’s steadfast predictive capability. The ROC curves for the 1, 3, and 5-year timeframes persistently reflected a noteworthy prognostic accuracy across an array of clinical and demographic subdivisions (Fig. 7A–F, Supplementary Fig. S3). Specifically, in patients below 65 years of age, the model yielded AUC values of 0.72, 0.75, and 0.78 for the 1, 3, and 5-year intervals, respectively. Conversely, for those aged 65 and above, the corresponding AUC values were 0.75, 0.78, and 0.77. The gender-disaggregated data further accentuated the model’s predictive finesse, with male patients exhibiting AUC values of 0.74, 0.82, and 0.81, and female patients showing 0.72, 0.69, and 0.74 for the same time intervals. The smoking status too played a discernible role; ‘Never Smoker’ category displayed AUC values of 0.87, 0.77, and 0.75, while the 'Ever Smoker & Current Smoker' category exhibited 0.71, 0.75, and 0.78. Transitioning to the supplementary findings, the prognostic signature continued to demonstrate substantial predictive precision in different pathological stages and classifications. In the Stage I–II category, the AUC values were 0.68, 0.76, and 0.76 for the 1, 3, and 5-year intervals, respectively (Supplementary Fig. S3). Similar trends were observed in different tumor (T) and node (N) stages, underlining the model’s consistent performance across varied clinical scenarios. Collectively, these detailed analyses underscore the robustness and the clinical relevance of our constructed prognostic model across a broad spectrum of patient demographics and clinical characteristics, establishing it as a potent tool for nuanced prognostic evaluation in LUAD.

### Risk signature associated with the tumour immune microenvironment in LUAD

**Figure 8:**
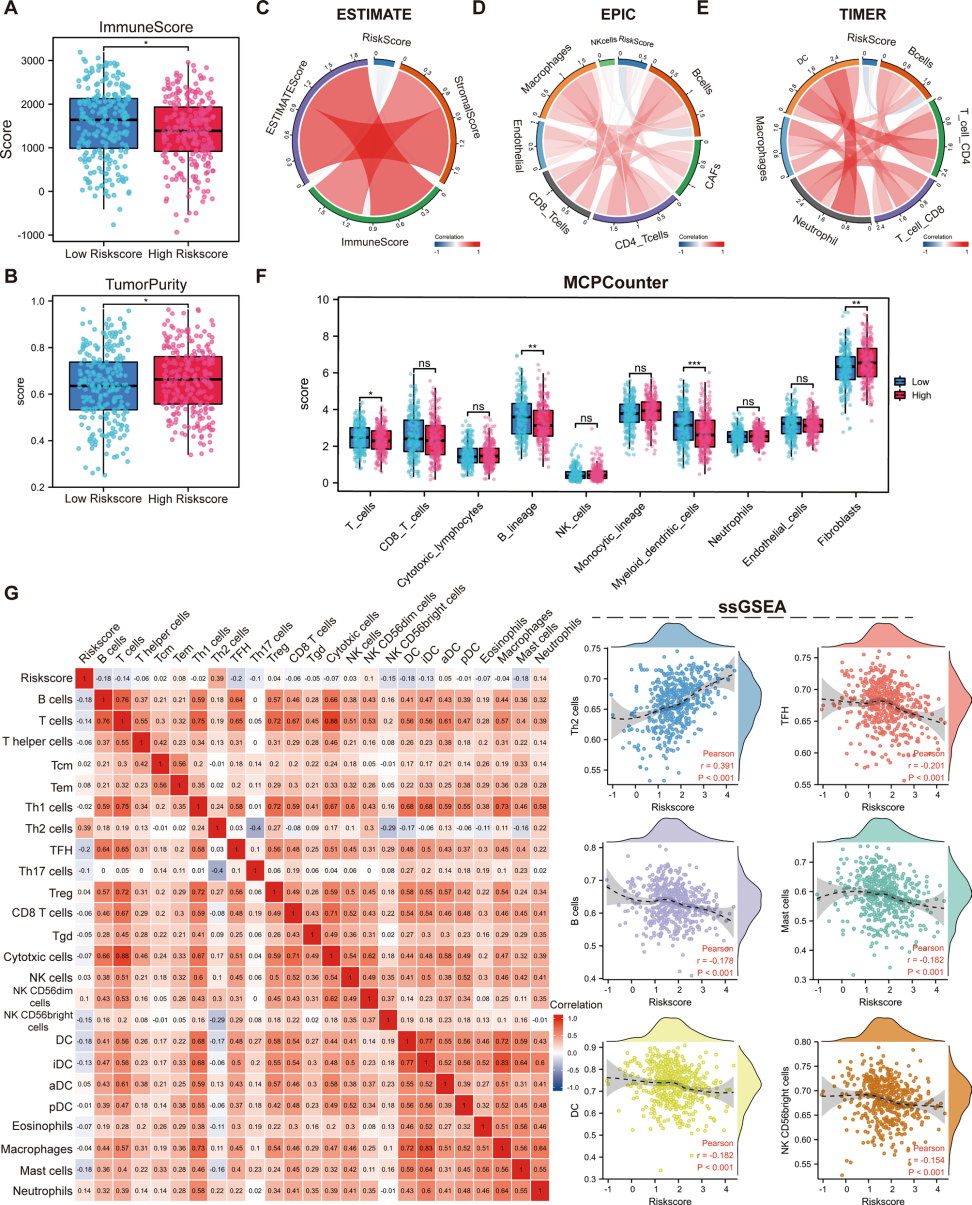
Dissecting the immune landscape interplay with endoplasmic reticulum stress-related signatures. (**A**, **B**) Comparative evaluation of ImmuneScore and TumorPurity metrics using the ESTIMATE algorithm, highlighting discrepancies between high- and low-risk groups. (**C**–**E**) Correlation plots showcasing the nuanced association between Risk scores and metrics of immune cell abundance or infiltration, as decoded by the ESTIMATE, EPIC, and TIMER algorithms. These plots accentuate the intricate ties between risk scores and the immunological realm. (**F**) A visual representation of the differential abundance of 10 pivotal immune cell types across the High- and Low- risk cohorts, deduced using the MCPCounter algorithm. This juxtaposition underscores the potential immunosuppressive tendencies of the high-risk group. (**G**) Relationship between Risk scores and abundance between 24 immune cell types.

In our quest to elucidate the intricate relationship between risk scores and immune cell subpopulations, we employed Spearman's correlation analysis. Launching our exploration with the ESTIMATE algorithm, we discerned discernible contrasts between the high- and low-risk score cohorts. Alarmingly, the high-risk group was characterized by a diminished ImmuneScore and an elevated TumourPurity (Fig. 8A,B). Branching out, we harnessed multiple immunization algorithms, casting a wider analytical net over the intricate interplay between risk scores and diverse immunization metrics. The ESTIMATE algorithm rang alarm bells, highlighting a negative affiliation between risk scores and parameters like ESTIMATEScore, StromalScore, and ImmuneScore. Complementarily, the EPIC and TIMER algorithms pointed towards a linkage between the risk score and these immune metrics. Venturing deeper into the tumor immune microenvironment, the MCPCounter algorithm was wielded to quantify the abundance of ten crucial immune cells (Fig. 8C–E). A landscape emerged where the high-risk group was laden with reduced abundance of pivotal cells like T cells, B Lineage, and Myeloid dendritic cells, while being saturated with fibroblasts (Fig. 8F). This painted a narrative where ERSRGs might be fostering tumor progression by curbing the antitumor immune system's spirited defense. Furthermore, ssGSEA analysis shed light on the intricate dance between risk scores and immune infiltration. A predominant negative correlation manifested between risk scores and a majority of the 24 immune cell types, including TFH, B cells, Mast cells, and more. However, a few immune cell types like Th2 cells and Neutrophils broke rank, showcasing a positive correlation (Fig. 8G). This intricate tapestry of interactions suggests that LUAD patients in the high-risk score bracket might be ensnared in an immune escape mechanism, a grim harbinger contributing to their adverse prognosis.

### Construction and validation of a predictive nomogram

**Figure 9:**
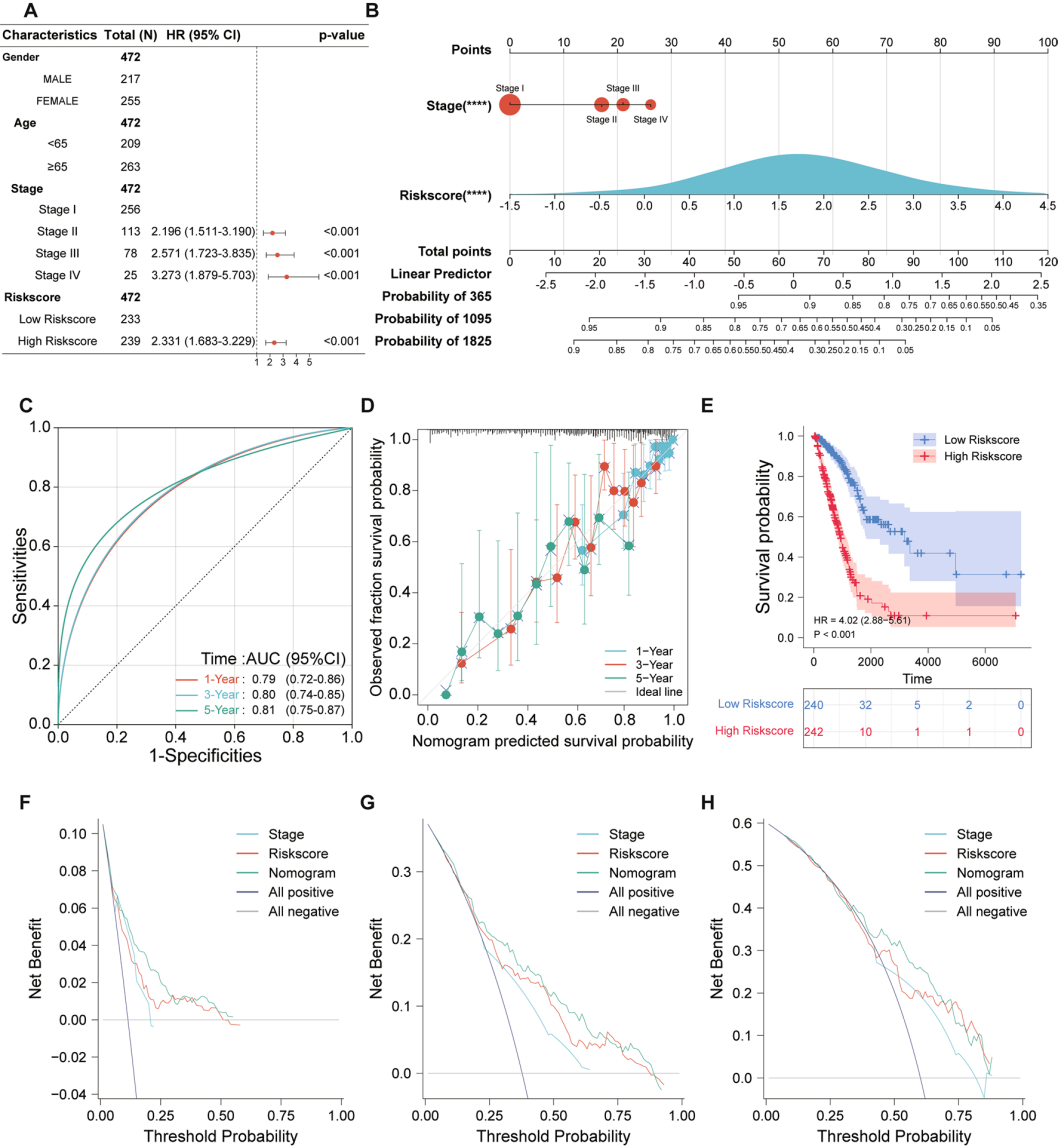
Comprehensive construction and validation of the prognostic nomogram. (**A**) Multivariate cox regression analysis highlighting the significance of clinical parameters: Gender, Age, Pathological Stages, and Risk score, in influencing LUAD prognosis. (**B**) A constructed predictive nomogram integrating the Risk score with Pathological Stages, offering clinicians a tangible tool to project 1-, 3-, and 5-year overall survival (OS) for LUAD patients. (**C**) ROC curves assessing the predictive precision of the nomogram across 1-, 3-, and 5-year OS intervals, with AUC values underscoring its reliability. (**D**) Calibration plot validating the nomogram's performance by comparing predicted versus observed 1-, 3-, and 5-year survival rates. (**E**) Kaplan–Meier survival curves, segregating patients based on the high and low risk scores derived from the nomogram, illustrating the differential survival outcomes between the two groups. (**F**–**H**) Decision curve analysis offering a comparative insight into the clinical utility of the nomogram versus other clinical parameters, specifically for forecasting 1-year (**F**), 3-year (**G**), and 5-year (**H**) OS.

To enhance the clinical applicability and translatability of our prognostic model, we embarked on the construction of a predictive nomogram utilizing the comprehensive dataset from TCGA. Our initial step was to discern the pivotal clinical parameters that would be integral to the nomogram. Employing multivariate Cox regression, we meticulously examined a suite of clinicopathological variables—spanning gender, age, pathological stages, and the risk score. This investigation spotlighted certain determinants, possessing marked significance (*p* < 0.001), which are depicted in Fig. 9A. With these insights, we architected a predictive nomogram, a synthesis of the pathological stages and the risk score, envisaged to provide clinicians with a tangible tool to prognosticate 1-, 3-, and 5-year OS for LUAD patients (Fig. 9B). To gauge the precision of our nomogram, we plotted ROC curves, which revealed encouraging AUC values: 0.79, 0.80, and 0.81 for the 1-, 3-, and 5-year OS intervals, respectively (Fig. 9C,D). Furthermore, the calibration curves, showcasing a promising alignment with the ideal diagonal, underscored the reliability of our nomogram (Fig. 9D). Validating this, our subsequent Kaplan-Meier survival analysis, which hinged on the risk delineation offered by the nomogram, corroborated our earlier findings—the high-risk cohort was plagued with a subpar prognosis (Fig. 9E). Lastly, we undertook a decision curve analysis. The insights were revelatory—the nomogram outshone both the pathological stages and the standalone risk score in its adeptness at forecasting the 1-, 3-, and 5-year overall survival trajectories for LUAD patients, a testament to its robustness (Fig. 9F–H).

### SELENOK inhibits migration and invasion of H1299 cells

**Figure 10:**
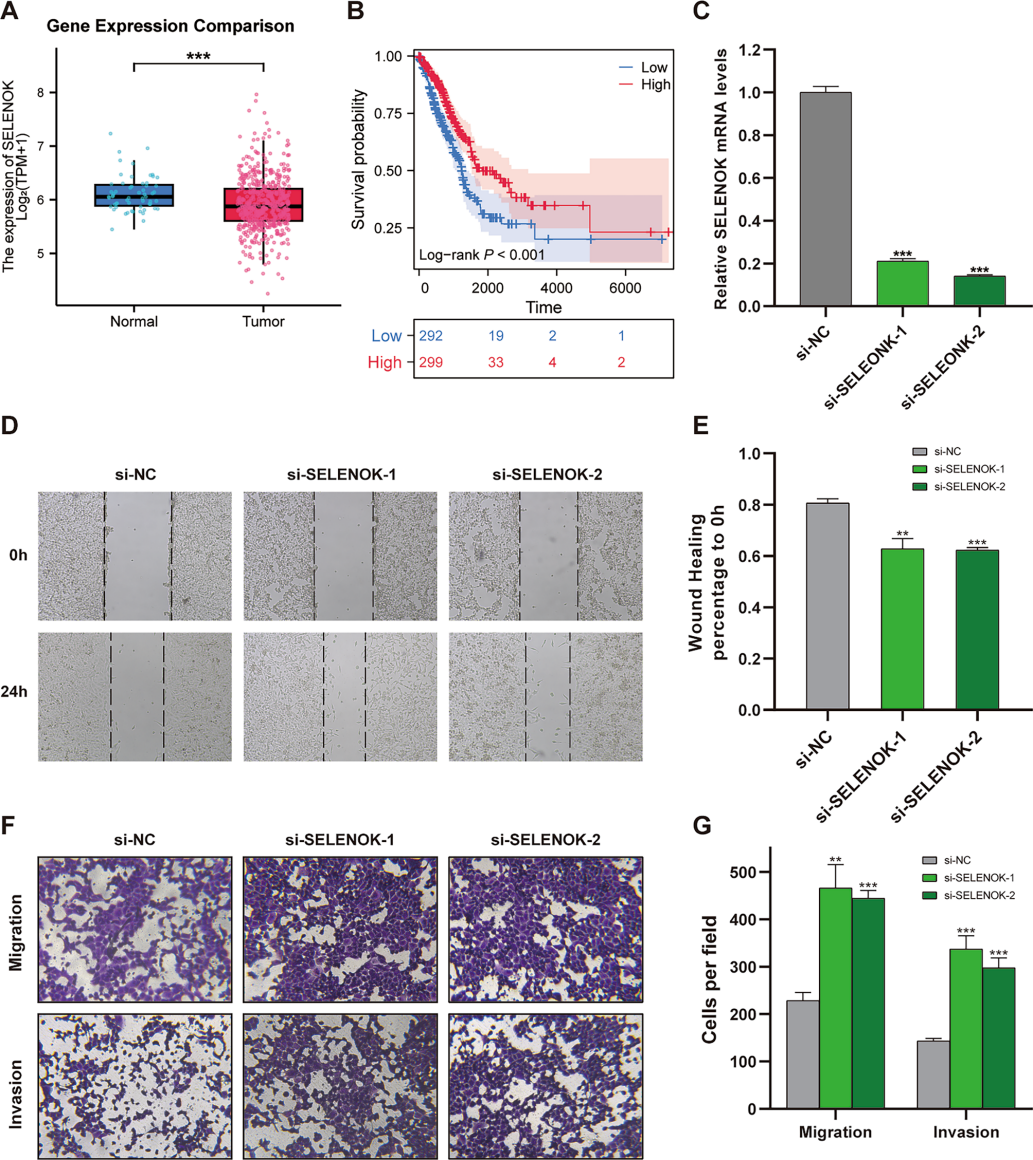
The effect of SELENOK on migration and invasion of H1299 cells. (**A**) Differential mRNA expression of SELENOK in adjacent normal tissues and tumor tissues in LUAD samples from TCGA database. (**B**) Analysis of the correlation between SELENOK expression levels and patient prognosis in LUAD dataset from TCGA database (*P* < 0.001). (**C**) RT-qPCR was used to detect changes in SELENOK mRNA expression in H1299 cells after SELENOK knockdown using siRNAs. Data are presented as mean ± standard deviation (n = 3). The difference between knockdown group and control group was compared using unpaired *t* test, ***P < 0.001. (**D**) H1299 cells transfected with si-NC, si-SELENOK-1, and si-SELENOK-2 were observed under an ordinary optical microscope at 0 h and 24 h after scratch, and representative images were randomly selected and displayed. (**E**) The scratch area of the randomly selected three fields was measured using ImageJ software, and the ratio of the area at 24 h to that at 0 h was calculated as the relative area of cell invasion into the scratch area. Data are presented as mean ± standard deviation (n = 3). The difference between overexpression group and control group was compared using unpaired *t* test, **P < 0.01, ***P < 0.001. (**F**) H1299 cells transfected with si-NC, si- SELENOK -1, and si- SELENOK-2, as well as corresponding control cells, were counted on a hemocytometer, and 20,000 H1299 cells were seeded into Transwell chambers for invasion assays, which required pre-coating with matrix gel. After 24 h, the migrated cells were stained, photographed, and observed under an ordinary optical microscope, and representative images were randomly selected and displayed. (**G**) The number of cells in the randomly selected fields was counted using ImageJ software, and data are presented as mean ± standard deviation (n = 3). The difference between overexpression group and control group was compared using unpaired t-test, **P < 0.01, ***P < 0.001.

Amid the spectrum of genes spotlighted in our earlier prognostic signature, SELENOK emerged as a particularly intriguing candidate, warranting a deeper dive into its functional implications in lung adenocarcinoma (LUAD). Initial forays into the TCGA database divulged a subdued expression profile of SELENOK in LUAD. Interestingly, an uptick in SELENOK expression correlated with a more favorable prognosis, hinting at its potential tumor-suppressive role in LUAD (Fig. 10A,B). To flesh out this hypothesis, a series of in-vitro experiments were undertaken. We engineered two tailored small interfering RNAs (siRNAs) to selectively quash SELENOK expression, with the resultant knockdown efficiency captured in Fig. 10C. The functional repercussions of this SELENOK silencing were assessed in the context of the migratory and invasive tendencies of H1299 cells, via scratch and Transwell assays, respectively (Figs 10D–G). Our observations painted a compelling picture: silencing SELENOK precipitated a marked decline in the migration and invasion capabilities of H1299 cells. This underscores SELENOK's pivotal role in curbing LUAD's invasive march. Collectively, these empirical findings not only bolster the credibility of our prior prognostic model but also spotlight SELENOK as a promising therapeutic target, offering fresh avenues for advancing LUAD prognosis and therapy.

## Discussion

Tumour heterogeneity and complexity are the key factors contributing to fatal outcomes, treatment failure and drug resistance in cancers, including lung cancer^13^. It is particularly important to identify new biomarkers of prognosis and develop more accurate prognostic models to predict the survival of patients with lung cancer to guide the treatment strategy. Previously reported prognostic models for ferroptosis-related genes, cell-cycle-checkpoints-related genes and aging-related-genes have exhibited promising results in predicting the prognosis in LUAD^14–16^. However, these biomarkers still have some limitations in terms of predictive power, and more accurate prognostic models are needed.

Previous studies suggested that ER stress is a rapid pathway for eukaryotic cells to respond to ER dysfunction, which results in the activation of the UPR^17,18^. In normal cells, the UPR response triggered by ER stress is often considered to be a form of cellular self-regulation that protects cells from irreversible damage^19,20^. In contrast, in cancer cells, the highly proliferative nature can activate the UPR response by disrupting the folding of ER proteins, thus allowing cancer cells to grow continuously in a nutrient-deprived environment^21^. The role of ER stress in lung cancer is still debated. IFN-γ is reported to promote apoptosis in tumour cells by increasing protein synthesis and triggering the UPR response^9^. Cisplatin can induce apoptosis in lung cancer cells through ER stress and autophagy^22^. Anti-cancer drug ABTL0812 can promote cancer cell death by inducing ER stress^23^. Some studies support the ability of ER Stress to inhibit cancer progression by promoting apoptosis; however, some studies are there are contradictory. Supplementation of PIMT under expression under ER stress promotes EMT and cell invasion in certain cell types of LUAD^12^. In addition, hypoxic environment, where cancer cells survive, leads to the UPR response resulting from ER stress induced by eIF2α phosphorylation, which is a necessary condition for hypoxic cell survival and tumour growth^24^. The multiplex status of ER stress in lung cancer suggests that it remains a valuable target in the treatment of malignant cancers. At the same time, ER Stress has a clear relevance to the tumour microenvironment. ER stress signalling can further regulate UPR-independent transcriptional and metabolic pathways in a cell-specific and context-dependent manner, thereby managing cellular phenotypes associated with cancer onset, progression and response or resistance to treatment^25^. Therefore, an intensive analysis of the biological features and clinical prognosis of ER stress and LUAD is still urgently needed.

In this study, we first identified 256 prognostic genes associated with ER stress in LUAD. We successfully classified 56 of these prognosis-related genes into 2 patterns, clusters 1 and 2. Cluster 2 exhibited a higher proportion of patients with LUAD with pathological stages III and IV, T stages T3 and T4, N stages N1 and N2, and M stage M1 and exhibited worse clinical features and prognosis compared with cluster 1. This clustering and its associated clinical features emphasize the potential utility of our ER Stress-related prognostic model in stratifying LUAD patients based on their disease severity and prognosis. Considering the close relationship between ER stress and immune microenvironment, a series of immune infiltration correlation analyses were performed^25,26^. Many immune cells in cluster 2, including B cells, T cells, TFH, Th17 cells and CD8^+^ T cells, exhibited lower expression. Based on these results, we can conclude that the immune status of cluster 2 promoted tumour progression and escape, whereas relative inhibition of tumor progression was observed in cluster 1. This is supported by the results of the ssGSEA algorithm analysis. Therefore, immunotherapy may be a better entry point for targeting cluster 2. To further analyse the functional characteristics of the two clusters at the biological functional level, enrichment analysis was performed for the two subtypes of differentially expressed genes using KEGG, GO and Hallmark’s 50 gene sets from the GSEA website. Cluster 2 was significantly enriched in cell-cycle-related signalling pathways such as E2F targets, G2M checkpoint, MYC targets, MTORC1 signalling, PI3K-AKTMTOR signalling, and p53 pathway^27–31^. The metastasis-related signalling pathways were also enriched, such as IL6/JAK/STAT3 signalling, and p53 pathway, TGF-β signaling, and Notch signaling^32–35^. Importantly, cluster 1 had a higher enrichment score for interferon α and interferon γ responses. The IFN family plays an important role in regulating and linking the innate and adaptive aspects of immunity. In addition, it plays obligate roles in the elimination phase of tumour immunoediting^36^. Previous studies demonstrated that IFN-γ-induced ER stress could trigger apoptosis in lung cancer cells^9^. Thus, manipulation of interferon signalling may be an effective immunotherapeutic tool for the treatment of cancer.

To further investigate the prognostic role of ER-stress-related genes on survival and treatment response, we undertook LASSO-Cox regression analysis and Multivariate Cox analysis. Drawing from 56 prognosis-related ER-stress-genes within the LUAD training set, a novel prognostic signature was fashioned, pivoting around 11 ER stress-associated genes, namely EIF2AK3, TMEM33, TTC23L, SELENOK, FBXO44, ERO1A, UGGT2, BCL2L1, TRAF2, ERLIN2, and PPP1R15B. This ER-stress-based prognostic signature elucidated that patients with elevated risk scores faced steeper mortality rates and a grimmer prognosis compared to their low-risk counterparts.

To gain a comprehensive perspective on the pathways and mechanisms instigated by these 11 genes, we consulted prior studies and illustrated our insights in a detailed molecular mechanism diagram (Supplementary Fig. 4). Genes like EIF2AK3, TMEM33, and SELENOK were spotlighted for their intrinsic roles in triggering or modulating ER stress^37,38^. Conversely, ERO1A and UGGT2 were delineated for their downstream involvement, either amplifying cell death avenues or fortifying cellular resilience against ER stress^39–44^.

Further validation of our prognostic signature was undertaken across diverse datasets, including the validation set, the entire TCGA, and GSE131210. The findings mirrored those of the training set, lending credibility to our model. Time-dependent ROC curves showcased commendable predictive prowess. When we probed the signature's capacity to predict clinical characteristics, clear disparities in clinical characteristics and prognosis emerged between the high- and low-risk groups. Those in the high-risk bracket consistently displayed a worse prognosis and a greater prevalence of adverse clinical characteristics. This was further substantiated by the time-dependent ROC curves, affirming the signature's predictive acumen.

We further analysed the relationship with the tumour immune microenvironment based on the prognostic signature constructed using ER-stress-related genes. Using the ESTIMATE, EPIC, TIMER and MCPCounter algorithms, we concluded that high-risk scores correspond to low immune infiltration, whereas low-risk scores are associated with high immune infiltration. This negative correlation suggested that patients with LUAD with high-risk scores are more likely to suffer from tumour immune escape.

To further enhance the precision of the prognostic signature, further calibration was conducted based on the prognostic risk score along with clinical features. Based on a multifactorial analysis of several clinical characteristics, a nomogram model was developed for the risk score in association with the pathological stage. The model was evaluated by time-dependent ROC curves and calibration curves. Moreover, decision curve analysis indicated the substantial clinical application of the nomogram model, which further supports the reliability of the signature.

Lung adenocarcinoma, with its inherent tumor heterogeneity, presents formidable challenges in treatment outcomes, often culminating in resistance to therapeutic interventions. Within this intricate matrix, the dysregulation of endoplasmic reticulum (ER) stress plays a pivotal role, sculpting the molecular landscape and trajectory of tumor progression. While ER stress in normal cells operates as a guardian, ensuring cellular integrity, its aberrant modulation in cancer cells potentially grants them an adaptive advantage, enabling relentless growth even in resource-scarce environments. This dynamic is further complicated by its interplay with immune signaling pathways, especially interferons, manifesting a dichotomy where it could either suppress or inadvertently advance tumor survival. Our constructed prognostic model, anchored around 11 salient genes tied to ER stress, offers a promising avenue for clinical stratification, pinpointing the severity and probable outcome of the disease with precision. Given the intricate relationship between ER stress and immune signaling, manipulating these pathways opens new pharmacological frontiers in cancer therapy. For instance, strategies revolving around modulating interferon signaling, amplifying or attenuating ER stress, or harnessing our ER stress-related prognostic markers could pave the way for more targeted and efficacious therapeutic regimens for patients with lung adenocarcinoma.

## Conclusions

In the current study, we divided LUAD patients into two molecular subtypes based on Endoplasmic Reticulum Stress associated prognostic genes in LUAD patients by consensus clustering. Functional and immunological analyses revealed that the unfolded protein reaction caused by endoplasmic reticulum stress dysregulation would affect the tumour microenvironment and ultimately lead to poor prognosis in lung adenocarcinoma patients. In addition, we constructed an Endoplasmic Reticulum Stress-related prognostic signature. The signature is a prognostic model consisting of a combined analysis of 11 ERSRGs, resulting in a prediction of clinical characteristics, prognosis, to be used as a clinically meaningful evaluation indicator. Our work could contribute to the risk stratification of lung cancer patients at a new dimension, thus providing ideas for new targeted drugs and theoretical guidance for personalised precision medicine.

## Methods

### Data collection and processing

The expression profiles [log2(TPM+1)] and clinical data for LUAD patients were downloaded from the The Cancer Genome Atlas (TCGA) database (https://portal.gdc.cancer.gov/). 490 tumor samples were obtained after standardisation and removal of samples with survival times of less than 30 days. GSE131210 was downloaded from the Gene Expression Omnibus (GEO) database (https://www.ncbi. nlm.nih.gov/geo/). After all samples with missing survival data were removed, the data were normalised and corrected to log2(x+1). Endoplasmic Reticulum Stress Related Genes originated from the GOBP_RESPONSE_TO_ of the GSEA MSIGDB database (http://www.gsea-msigdb.org/gsea/index.jsp) ENDOPLASMIC_RETICULUM_STRESS dataset with 256 genes. The gene expression differences were analysed using Limma (linear models for microarray data), and we used the R package "limma" (version 3.40.6) for Differentially expressed genes (DEGs) analysis (|FoldChange| > 2, adjusted *p* < 0.05)^45^.

### Consensus clustering and molecular subtypes of endoplasmic reticulum stress associated genes

The clustering analysis used ConsensusClusterPlus: a class discovery tool with confidence assessment and item tracking, using agglomerative km clustering with a euclidean distance and resampling 80% of the samples for 1000 repetitions^46^. The optimal number of clusters was determined by using the empirical cumulative distribution function plot. In addition, the R package "scatterplot3d" was used to perform multidimensional cluster analysis to evaluate the clustering efficiency.

### Tumour immune microenvironment

In this study, we used the Immuno-Oncology Biological Research (IOBR) tool to perform immuno-tumor biology calculations^47^. The immune infiltrating cell score of the samples was calculated based on the expression profiles using ESTIMATE, TIMER, EPIC, MCPCounter algorithms and R package ‘IOBR’^48–52^. The ssGSEA algorithm was used to calculate the infiltration level of 24 types of immune cells^53^.

### Functional enrichment analysis

The c2.cp.kegg.v7.4.symbols.gmt (KEGG) and h.all.v7.4.symbols.gmt (Hallmark) gene sets were downloaded from the Molecular Signatures Database (http://www.gsea-msigdb.org/gsea/msigdb/index.jsp)^54–57^. Gene Set Variation Analysis (GSVA) was performed using the R package ‘GSVA’, which calculates the enrichment fraction of each sample in the Hallmark gene set, setting the minimum set size to 5 and the maximum set size to 5000^58^.

### Construction of the prognostic signature

After integrating survival outcomes, survival times, and gene expression profiles, we performed regression analysis using the R package ‘glmnet’ and LASSO-Cox and obtained the best signature by 10-fold cross validation^59,60^. We performed a Multivariate stepwise regression analysis on the LASSO results using the R package "survival"^61^. Finally, a prognostic signature consisting of 11 ER Stress genes was obtained. The signature is calculated as follows:

$${\text{Risk score }} = \, \left[ {{\text{Coef}}\left( {1} \right) \, \times {\text{ gene Exp}}\left( {1} \right)} \right] \, + \, \left[ {{\text{Coef}}\left( {2} \right) \, \times {\text{ gene Exp}}\left( {2} \right)} \right] \, + \, ...... \, + \, \left[ {{\text{Coef}}\left( {\text{i}} \right) \, \times {\text{ geneExp}}\left( {\text{i}} \right)} \right]$$

^62,63^.

Kaplan–Meier analysis was performed using the R package ‘survival’ and ‘survfit’ functions, and the log rank test was used to assess the significance of prognostic differences between different groups of LUAD samples to examine the predictive efficiency and applicability of the signature. In addition, receiver operating characteristic (ROC) analysis was performed using the R package ‘pROC’ to obtain AUCs to further validate the applicability and efficiency of the signature. R package ‘rms’ was used to construct a COX regression nomogram to assess the prognostic value of clinicopathological factors and risk scores in the LUAD sample^64^.

### Cell lines and cell culture

Human lung adenocarcinoma (LUAD) cell lines A549 and H1299 were derived from the Cell Bank of the Chinese Academy of Sciences and cultured using RPMI 1640 medium (HyClone, South Logan, UT. USA) and 10% fetal bovine serum (FBS; Invitrogen, Carlsbad, CA, USA) at 37 °C in a 5% CO_2_ humidified incubator.

### Wound-healing migration assay

H1299 cells were grown in six-well plates until they formed a 90% confluent monolayer. Then, a sterile pipette tip was used to scratch the cells and they were cultured in serum-free medium for 24 h. The cells were then photographed in three random fields under a microscope and the distance of cell migration into the scratched area was measured using ImageJ Launcher software. This analysis was repeated in triplicate.

### Transwell migration and invasion assays

20000 H1299 cells were added to the upper chamber of Transwell plates with 200 μl of RPMI 1640 medium containing 1% FBS. The lower chamber contained 800 μl of RPMI 1640 medium with 10% FBS as a chemoattractant. After incubation for 24 h at 37 °C, the inserts were removed and the non-migrating or non-invading cells were removed with cotton swabs. The migrating or invading cells that reached the lower chamber were fixed, stained with 1% crystal violet, photographed, and counted in at least three random fields. Each Transwell migration and invasion analysis was performed in triplicate.

### Supplementary Information

Supplementary Figure 1.

Supplementary Figure 2.

Supplementary Figure 3.

Supplementary Figure 4.

## Data Availability

The datasets generated and analysed during the current study are available in the TCGA database (https://portal.gdc.cancer.gov/), GSEA MSIGDB database (http://www.gsea-msigdb.org/gsea/index.jsp) and GEO database (https://www.ncbi.nlm.nih.gov/geo/).
